# Jasmonate signaling is activated in the very early stages of iron deficiency responses in rice roots

**DOI:** 10.1007/s11103-016-0486-3

**Published:** 2016-05-03

**Authors:** Takanori Kobayashi, Reiko Nakanishi Itai, Takeshi Senoura, Takaya Oikawa, Yasuhiro Ishimaru, Minoru Ueda, Hiromi Nakanishi, Naoko K. Nishizawa

**Affiliations:** Japan Science and Technology Agency, PRESTO, 4-1-8 Honcho, Kawaguchi, Saitama 332-0012 Japan; Research Institute for Bioresources and Biotechnology, Ishikawa Prefectural University, 1-308 Suematsu, Nonoichi, Ishikawa 921-8836 Japan; Graduate School of Agricultural and Life Sciences, The University of Tokyo, 1-1-1 Yayoi, Bunkyo-ku, Tokyo, 113-8657 Japan; Graduate School of Science, Tohoku University, 6-3 Aramaki-aza Aoba, Aoba-ku, Sendai, 980-8578 Japan

**Keywords:** HRZ, IDEF1, Iron deficiency, Jasmonates, Rice, Ubiquitin ligase

## Abstract

Under low iron availability, plants induce the expression of various genes involved in iron uptake and translocation at the transcriptional level. This iron deficiency response is affected by various plant hormones, but the roles of jasmonates in this response are not well-known. We investigated the involvement of jasmonates in rice iron deficiency responses. High rates of jasmonate-inducible genes were induced during the very early stages of iron deficiency treatment in rice roots. Many jasmonate-inducible genes were also negatively regulated by the ubiquitin ligases OsHRZ1 and OsHRZ2 and positively regulated by the transcription factor IDEF1. Ten out of 35 genes involved in jasmonate biosynthesis and signaling were rapidly induced at 3 h of iron deficiency treatment, and this induction preceded that of known iron deficiency-inducible genes involved in iron uptake and translocation. Twelve genes involved in jasmonate biosynthesis and signaling were also upregulated in *HRZ*-knockdown roots. Endogenous concentrations of jasmonic acid and jasmonoyl isoleucine tended to be rapidly increased in roots in response to iron deficiency treatment, whereas these concentrations were higher in *HRZ*-knockdown roots under iron-sufficient conditions. Analysis of the jasmonate-deficient *cpm2* mutant revealed that jasmonates repress the expression of many iron deficiency-inducible genes involved in iron uptake and translocation under iron sufficiency, but this repression is partly canceled under an early stage of iron deficiency. These results indicate that jasmonate signaling is activated during the very early stages of iron deficiency, which is partly regulated by IDEF1 and OsHRZs.

**Electronic supplementary material:**

The online version of this article (doi:10.1007/s11103-016-0486-3) contains supplementary material, which is available to authorized users.

## Introduction

Iron (Fe) is an essential element for virtually all living organisms. Plants take up Fe from the rhizosphere, where it is abundant but only slightly soluble. Fe solubility and thus availability is especially low in calcareous soils, which cover one-third of the world’s cultivated areas, severely limiting plant productivity. Therefore, understanding plant responses to low Fe availability is important for breeding crops with better growth under adverse conditions.

In response to low Fe availability, plants induce the expression of genes involved in Fe uptake. This response is categorized into two strategies in higher plants. Non-graminaceous plants utilize the reduction strategy (Strategy I), while graminaceous plants utilize the chelation strategy (Strategy II) (Römheld and Marschner 1986). Strategy I is characterized by induction of ferric-chelate reductase FRO at the root surface and subsequent uptake of generated Fe^2+^ ions by induced IRT transporters (Eide et al. 1996; Robinson et al. 1999; Vert et al. 2002). This Fe^2+^ uptake is supported by the secretion of protons and phenolic compounds by HA and ABCG/PDR transporters, respectively (Santi and Schmidt 2009; Rodríguez-Celma et al. 2013; Fourcroy et al. 2014; Schmid et al. 2014). Strategy II is mediated by Fe(III) chelators biosynthesized in graminaceous plants, designated mugineic acid family phytosiderophores (MAs) (Takagi 1976). MAs are secreted to the rhizosphere by TOM1 transporter and chelate Fe(III) (Nozoye et al. 2011). Subsequently, the resulting Fe(III)-MAs complexes are absorbed into the roots by YS1/YSL transporters (Curie et al. 2001, 2009; Murata et al. 2006; Inoue et al. 2009). These two strategies have been previously considered mutually exclusive, but some exceptions have been reported recently. For example, rice, a graminaceous plant, biosynthesizes 2′-deoximugineic acid (DMA) among MAs and takes up Fe(III)-DMA as a Strategy II mechanism, but it also takes up Fe^2+^ by OsIRT1 transporter (Ishimaru et al. 2006). Meanwhile, peanut, a Strategy I plant, also utilizes Fe(III)-MAs when MAs are supplied from the intercropped grasses (Xiong et al. 2013a).

In both strategies, strong induction of the corresponding enzymes and transporters takes place at the transcript level in response to low Fe availability (Kobayashi and Nishizawa 2012). In *Arabidopsis thaliana*, a Strategy I plant, this response is primarily mediated by basic helix-loop-helix (bHLH) transcription factors FIT and subgroup Ib bHLHs (Colangelo and Guerinot 2004; Jakoby et al. 2004; Yuan et al. 2005, 2008; Sivitz et al. 2012; Wang et al. 2013b). Fe deficiency-inducible gene expression in *Arabidopsis* is also regulated by PYE and subgroup IVc bHLH transcription factors, and by MYB transcription factors MYB10 and MYB72 (Long et al. 2010; Palmer et al. 2013; Zhang et al. 2015). In rice, the genes involved in Fe(III)-DMA uptake, Fe^2+^ uptake and Fe translocation are differentially regulated by various transcription factors (Kobayashi et al. 2014). The genes involved in DMA-based Fe uptake are regulated by the bHLH transcription factors OsIRO2 and OsIRO3, and their upstream transcription factor IDEF1 (Ogo et al. 2007, 2011; Kobayashi et al. 2007, 2009, 2014; Zheng et al. 2010). The Fe^2+^ transporter gene *OsIRT1* is regulated by IDEF1 and OsIRO3 (Kobayashi et al. 2007, 2009; Zheng et al. 2010). Fe translocation within the plant is regulated mainly by the transcription factors IDEF1, IDEF2 and OsbHLH133 (Kobayashi et al. 2007, 2009; Ogo et al. 2008; Wang et al. 2013a). Among the above-mentioned *Arabidopsis* and rice transcription factors, all but IDEF1, IDEF2 and subgroup IVc bHLHs are transcriptionally induced under Fe deficiency.

In addition to the specific genes involved in Fe uptake, translocation and their regulation, numerous other genes are transcriptionally induced under Fe-deficient conditions, as characterized by transcriptomic analyses (Schmidt and Buckhout 2011; Kobayashi et al. 2014). In contrast, knowledge of protein-level expression responses under Fe deficiency remains limited. A recent proteome analysis revealed that the Fe deficiency responses are conserved only partially between the transcript and protein levels in *Arabidopsis* (Lan et al. 2011). Protein modification and degradation also play important roles in plant responses to environmental fluctuations. In *Arabidopsis*, subcellular localization, vacuolar sorting and degradation of Fe^2+^ transporter IRT1 are controlled by monoubiquitination mediated by a RING ubiquitin ligase IDF1 (Barberon et al. 2011; Shin et al. 2013). The *Arabidopsis* transcription factor FIT, a central regulator of the Strategy I response, is subjected to degradation via the 26S proteasome pathway mediated by unknown E3 ubiquitin ligases (Lingam et al. 2011; Sivitz et al. 2011). This degradation is inhibited by interaction with EIN3 and EIL1, which play central roles in ethylene signaling (Lingam et al. 2011) and is enhanced under Fe-deficient conditions (Sivitz et al. 2011). Two *Arabidopsis* subgroup IVc bHLH transcription factors, ILR3 and AtbHLH115, are bound to and destabilized by a RING ubiquitin ligase, BTS (Selote et al. 2015). ILR3 and AtbHLH115 also bind to transcription factor PYE (Long et al. 2010). *pye* knockout mutants are susceptible to Fe deficiency, whereas *bts* knockdown mutants are tolerant to Fe deficiency (Long et al. 2010; Zhang et al. 2015), suggesting antagonism between PYE and BTS functions. BTS binds to Fe and zinc (Zn) (Kobayashi et al. 2013) and is destabilized by Fe in in vitro translation reactions (Selote et al. 2015), suggesting its role as an intracellular Fe sensor. Rice IDEF1, a central transcriptional regulator of Fe deficiency responses, also binds to Fe^2+^ and other divalent metals, and is proposed to also be a Fe sensor (Kobayashi et al. 2012). IDEF1 is subjected to 26S proteasome-mediated degradation, which is likely inhibited by interaction with the Bowman-Birk trypsin inhibitor IBP1.1 and possibly by its close homolog IBP1.2 (Zhang et al. 2014). E3 ubiquitin ligases involved in modification or degradation of proteins involved in Fe deficiency responses in graminaceous plants have not been reported.

Previously, we identified two rice RING ubiquitin ligases, OsHRZ1 and OsHRZ2, which bind to Fe and Zn and negatively regulate Fe deficiency responses (Kobayashi et al. 2013). OsHRZ1 and OsHRZ2 are close homologs of *Arabidopsis* BTS and similar proteins are widely present in higher plants and algae (Urzica et al. 2012; Kobayashi et al. 2013), suggesting the conservation of HRZs/BTS-type putative Fe sensors in the plant kingdom. Knockdown mutants of *OsHRZ1* and *OsHRZ2* showed improved tolerance to Fe deficiency and accumulation of Fe and Zn in shoots and seeds irrespectively of Fe nutritional conditions (Kobayashi et al. 2013). Thus, knockdown of HRZs is a promising method of producing Fe- and Zn-fortified crops with improved growth under Fe-limiting conditions. Microarray and quantitative RT-PCR analyses showed that expression of the majority of known Fe deficiency-inducible genes involved in Fe uptake and translocation is enhanced in these *HRZ*-knockdown lines, especially under Fe-sufficient conditions. OsHRZ1 and OsHRZ2 possess ubiquitination activity in vitro, but their substrate proteins have not been identified. In addition to the RING Zn-finger domain which mediates ubiquitination, OsHRZ1 and OsHRZ2 possess two additional Zn-finger domains, suggesting potential roles in transcriptional, post-transcriptional or translational regulation.

Various plant hormones and small signaling molecules such as nitric oxide also regulate Fe deficiency responses. Auxin, ethylene and nitric oxide positively regulate Fe deficiency responses in both graminaceous and non-graminaceous plants (Hindt and Guerinot 2012; Kobayashi and Nishizawa 2012). These three molecules are thought to affect each other and are required for Fe deficiency responses (García et al. 2011; Hindt and Guerinot 2012). In *Arabidopsis*, abscisic acid and gibberellin are also suggested to play positive roles in Fe deficiency responses (Lei et al. 2014; Matsuoka et al. 2014), whereas cytokinin and jasmonic acid (JA) act negatively (Séguéla et al. 2008; Maurer et al. 2011). Brassinosteroids negatively regulate the expression of genes involved in Fe uptake and translocation in cucumber and rice (Wang et al. 2012, 2015). Abscisic acid could potentially share the signaling pathway with IDEF1-mediated Fe deficiency responses in rice (Kobayashi et al. 2007, 2009). The roles of gibberellin, cytokinin, and JA in Fe deficiency responses in graminaceous plants remain unclear.

JA and its derivatives, jasmonates (JAs), are oxylipin-based plant hormones rapidly synthesized in response to various stresses, such as wounding and pathogens, subsequently mediating the defense mechanisms against these stimuli (Fonseca et al. 2009; Vleesschauwer et al. 2013). JAs are sensed by the SCF^COI1^ complex of E3 ubiquitin ligase, which targets JAZ repressors for 26S proteasome-mediated degradation. As a consequence, the bHLH transcription factor MYC2/JIN1 is de-repressed, mediating the expression of JA-regulated genes in the presence of JAs. Studies on the JA receptor COI1 revealed the bioactive component of JA to be jasmonoyl isoleucine (JA-Ile) (Fonseca et al. 2009). Although there is no report on JA function in Fe deficiency responses, with the exception of repressive roles in *Arabidopsis* (Maurer et al. 2011), recent findings suggest positive involvement of JAs in Fe deficiency responses in rice. Firstly, OsRMC, a JA-induced receptor-like protein, negatively regulates JA-mediated root development (Jiang et al. 2007), and positively regulates Fe deficiency-inducible genes in rice (Yang et al. 2013). Secondly, two IDEF1-binding Bowman-Birk trypsin inhibitors IBP1.1 and IBP1.2 that putatively mediate Fe deficiency responses via IDEF1 (Zhang et al. 2014) are transcriptionally induced by JA treatment (Yoshii et al. 2010). Lastly, two close homologs of HRZ ubiquitin ligases, LjnsRING in *Lotus japonicus* and TARF in tobacco, regulate infection by symbiotic bacteria and tobacco mosaic virus, respectively (Shimomura et al. 2006; Yamaji et al. 2010), suggesting possible involvement in JA-mediated defense responses.

In the present study, we investigated whether the JA pathway is involved in rice Fe deficiency responses. Expression analysis revealed that the JA pathway is rapidly activated during the early stages of Fe deficiency in rice roots, and that this pathway is regulated by OsHRZs and IDEF1. Endogenous concentrations of JAs tended to be increased in rice roots under early Fe deficiency, and were higher in *HRZ*-knockdown roots under Fe sufficiency. Analysis of a JA-deficiency mutant revealed that JAs regulate the genes involved in Fe uptake and translocation dependent on Fe nutritional status. These results revealed a positive involvement of the JA pathway during the early stages of Fe deficiency in rice roots.

## Materials and methods

### Analysis of the transcriptional relationship between JA and Fe deficiency responses

The corresponding clones of the JA-responsive genes reported previously by Yoshii et al. (2010) and Seo et al. (2011) were identified on the rice 44 K microarray slide (Agilent Technologies, USA). Based on the summation of microarray and RNA gel blot analyses of suspension-cultured non-transgenic rice after treatment with 50 μM JA for 1 and 6 h (Yoshii et al. 2010) and microarray analysis of 2-wk-old non-transgenic rice seedlings after treatment with 100 μM methyl-JA for 6 h (Seo et al. 2011), 109 and 29 clones were chosen as early JA-induced and JA-repressed genes, respectively. Based on the microarray analysis of non-transgenic rice after treatment with 30 μM JA for 12 days (Yoshii et al. 2010), 287 and 71 clones were chosen as late JA-induced and JA-repressed genes, respectively. Genes involved in JA biosynthesis and signaling were derived from the description by Hirano et al. (2008), except for OsCOI1a and OsCOI1b which were derived from the description by Yang et al. (2012).

Transcriptional early Fe deficiency responses for the 3–36-h treatments in roots were based on a previous microarray analysis by Itai et al. (2013). Transcripts showing log_2_ expression ratios >1 and <−1 were grouped as induced and repressed transcripts, respectively, only if they showed a signal value >20 and significance by *t* test within an array slide (*P* < 0.05). Transcriptional responses to 7 days of Fe deficiency in roots were based on a previous microarray analysis by Ogo et al. (2008). Transcripts with a signal value >100 and *P* < 0.05 showing log_2_ expression ratios >1 and <−1 were grouped as induced and repressed transcripts, respectively. Transcriptional responses to 6 or 24 h of nitrogen (N), phosphorus (P) or potassium (K) deficiency in roots were based on a previous microarray analysis and threshold (false discovery rate <0.05 and fold change >2.0) described by Takehisa et al. (2013). Transcriptional responses in the *HRZ*-knockdown roots (which show slight repression of *OsHRZ1* expression and stronger repression of *OsHRZ2* expression) were evaluated based on Kobayashi et al. (2013). Transcripts with a signal value >20 and *P* < 0.05 showing average log_2_ expression ratios for line 2i-2/non-transformant and line 2i-3/non-transformant >1 were grouped as induced transcripts. Transcriptional responses to *IDEF1* induction in roots were based on a previous microarray analysis and threshold described by Kobayashi et al. (2009). Transcripts with a signal value >100 and *P* < 0.05 showing log_2_ expression ratios for the *IDEF1* induction line 13/non-transformant >1 were grouped as induced transcripts. *Cis*-sequence distribution analysis was performed according to Ogo et al. (2008) and Kobayashi et al. (2009). Significant overrepresentation or underrepresentation compared with the average rate for all 42,123 clones was analyzed using a binomial distribution test. Transcriptional responses of Fe deficiency-related genes to 100 μM JA treatment in roots were derived from the data provided on the RiceXPro website (Sato et al. 2013; http://ricexpro.dna.affrc.go.jp/index.html).

### Plant materials and growth conditions

Non-transgenic rice (*Oryza sativa* L. cultivar Tsukinohikari) was germinated on Murashige and Skoog medium, while the *HRZ*-knockdown line 2i-3 (Kobayashi et al. 2013) was germinated on Murashige and Skoog medium with hygromycin B (50 mg/L). After 18 days of culture followed by 3 days of acclimation, the plantlets were transferred to a hydroponic solution in a greenhouse under a 30 °C light and 25 °C dark cycle with natural light conditions. The composition of the hydroponic solution was as follows: 0.70 mM K_2_SO_4_, 0.10 mM KCl, 0.10 mM KH_2_PO_4_, 2.0 mM Ca(NO_3_)_2_, 0.50 mM MgSO_4_, 10 μM H_3_BO_3_, 0.50 μM MnSO_4_, 0.50 μM ZnSO_4_, 0.20 μM CuSO_4_, 0.01 μM (NH_4_)_6_Mo_7_O_24_, and 100 μM Fe(III)–EDTA. The pH of the nutrient solution was adjusted to 5.5 every 2–3 days. After 10 days of preculture in hydroponic solution, Fe deficiency was initiated by omitting Fe(III)-EDTA from the solution at 10:30 AM. Fe sufficiency (control) plants were cultured similarly with Fe(III)-EDTA. Whole roots were harvested from two plants per replicate at 6 and 24 h after initiating the treatments, immediately frozen in liquid nitrogen and used for further analyses.

The JA-deficient *cpm2* mutant (Riemann et al. 2013) was kindly provided by Dr. Moritoshi Iino (Osaka City University, Japan), Dr. Hisakazu Yamane, Dr. Koji Miyamoto (Teikyo University, Japan) and Dr. Kazunori Okada (The University of Tokyo, Japan). Wild-type rice (cultivar Nihonmasari) and *cpm2* mutant were germinated on Murashige and Skoog medium. Homozygous *cpm2* mutants were selected by long coleoptile phenotype (Riemann et al. 2013) and confirmed by genomic PCR. Hydroponic culture was carried out as above, except that the preculture in hydroponic solution was shortened to 5 days and Fe-deficiency treatment was extended to 7 days. Relative chlorophyll content of the youngest leaf was measured using a SPAD-502 chlorophyll meter (Konica Minolta, Tokyo, Japan). Hydroponic solution was renewed at 4 days after initiating the treatments. For quantitative RT-PCR analysis, whole roots were harvested at 24 h after initiating the treatments.

### Quantification of endogenous JAs

Frozen roots (30–200 mg fresh weight) were ground into a powder using a mortar and pestle, and total JAs were extracted with 1 mL ethanol. After an overnight incubation at 4 °C in the dark followed by centrifugation at 20,000×*g* for 5 min, 900 μL of the supernatant were transferred to a new tube, and the liquid was evaporated by vacuum at room temperature. The extract was resuspended in 40 μL ultrapure water and centrifuged at 20,000×*g* for 20 min. A 35-μL aliquot was transferred to a new tube and centrifuged again at 20,000×*g* for 20 m, after which 30 μL were transferred to a 0.8-mL Sn-Vial (GL-Science, Japan), and 10 µL of sample with internal standards were subjected to ultra-performance liquid chromatography coupled to time-of-flight mass spectrometry (UPLC/TOFMS) analysis (Sato et al. 2009). UPLC/TOFMS analysis was performed using an Agilent 1290 Infinity (Agilent Technologies) coupled to a micrOTOF II (Bruker Daltonics, Germany). A ZORBAX Eclipse Plus C18 column (1.8 µm, 2.1 × 50 mm; Agilent Technologies) was used to separate the compounds. The mobile phases were A, 20 % (v/v) aqueous methanol with 0.05 % (v/v) acetic acid, and B, methanol with 0.05 % (v/v) acetic acid. The gradient program was 0–3.5 min, isocratic 90 % A; 3.5–6 min, linear gradient 90–0 % A; 6.1–9 min, isocratic 90 % A, with a flow rate of 0.15 mL/min. The mass spectrometer was run in the negative mode with a scan range of 100–700 *m/z*. The capillary voltage was 4200 V, the nebulizer gas pressure was 1.6 bar, the desolvation gas flow was 8.0 L/min, and the temperature was 180 °C. JA and JA-Ile were quantified on the basis of an extracted ion chromatogram and the corresponding peak position of the standard solution.

### Quantitative RT-PCR analysis

Total RNA was extracted from roots using the RNeasy Plant Mini Kit (Qiagen), treated with DNase I and reverse-transcribed using ReverTra Ace qRT-PCR RT Master Mix with gDNA Remover (Toyobo). Real-time PCR was performed in a StepOnePlus™ Real-Time PCR System (Applied Biosystems) with SYBR Premix Ex Taq™ II (TaKaRa) or with TaqMan Gene Expression Assays (Applied Biosystems). The transcript abundance was normalized against the rice α-2 tubulin transcript level and was expressed as a ratio relative to the levels in the Fe-sufficient non-transgenic roots at 6 h or Fe-sufficient wild-type roots. Primers used for quantitative RT-PCR are shown in Online Resource 1.

## Results

### JA responses are activated during the very early stages of Fe deficiency

**Fig. 1:**
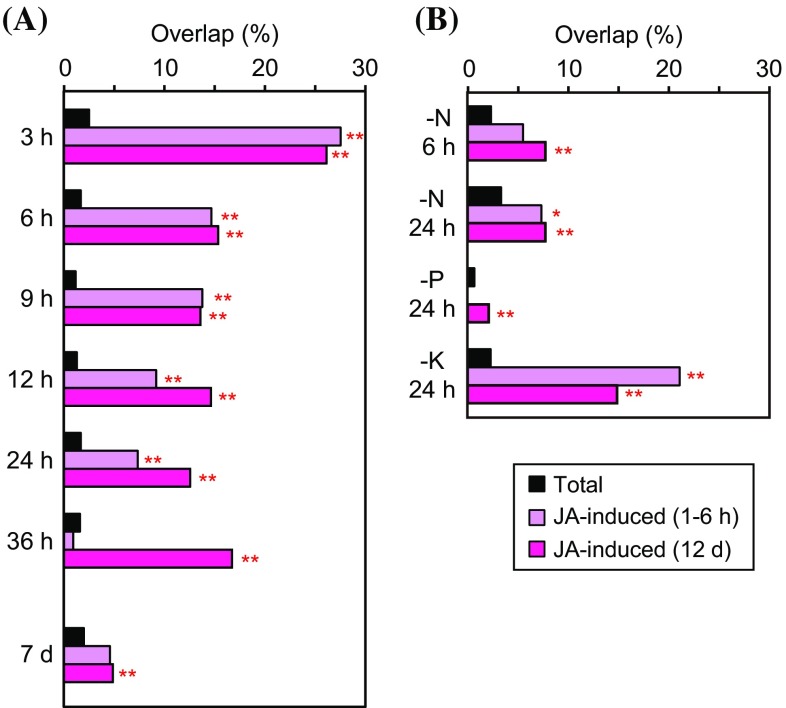
Overlap of the genes induced by jasmonates (JAs) with the nutrient deficiency-induced genes in roots. **a** Fe deficiency-induced genes. **b** N, P or K deficiency-induced genes. Genes induced by JA treatment after 1–6 h and 12 days (d) were analyzed based on previous reports by Yoshii et al. (2010) and Seo et al. (2011). Fe deficiency-induced genes at 3–36 h and 7 days are based on previous microarray analyses by Itai et al. (2013) and Ogo et al. (2008), respectively. N, P or K deficiency-induced genes at 6 or 24 h are based on previous microarray analysis by Takehisa et al. (2013). Overlap was calculated as (the number of the clones showing nutrient deficiency-induction and JA-induction)/(the number of the total clones showing JA-induction). “Total” rate was calculated as (the number of the total clones showing nutrient deficiency-induction)/(the number of the total clones analyzed). *Asterisks* indicate significant overrepresentations compared with the total rates (**P < 0.01). No significant underrepresentation was observed

Using the 44 K microarray platform, we investigated the expression patterns of genes previously reported to be induced after 1–6 h or 12 days of JA treatment (Yoshii et al. 2010; Seo et al. 2011). Overlaps of these JA-induced genes with those induced by Fe deficiency were calculated based on our previous microarray studies of early (3–36 h) and subsequent (7 days) Fe deficiency treatments in non-transgenic rice roots (Ogo et al. 2008; Itai et al. 2013) (Fig. 1a). We found notably high overlap of JA-induced genes with the Fe deficiency-induced genes at the early stages of treatment (Fig. 1a). Among the JA-induced genes at 1–6 h, 28 % was also induced at 3 h of Fe deficiency, and this overlap gradually decreased with the duration of Fe deficiency treatment until 36 h. The overlap of JA-induced genes at 12 days was also the highest at 3 h of Fe deficiency (26 %), but it remained high (13–15 %) throughout the 6–36 h of Fe deficiency treatment. Although this rate decreased to 5 % at 7 days of Fe deficiency, it was still overrepresented in comparison with the total rate of the Fe deficiency-induced genes at 7 days. These results strongly suggest that the JA responses are rapidly activated at the onset of Fe deficiency.

We also investigated the genes repressed by JA treatment reported by Yoshii et al. (2010) and Seo et al. (2011). The JA-repressed genes at 12 days, but not 1–6 h, constituted significantly high overlap with Fe deficiency-induced genes, especially during the early stages (Online Resource 2). We also found high overlap of JA-induced and JA-repressed genes with Fe deficiency-repressed genes, especially in the combinations of 12-days JA response and 7-days Fe deficiency response (Online Resource 2). These results suggest that both activating and repressive pathways involved in JA responses are linked to both activating and repressive regulation under Fe deficiency.

In order to estimate specificity of the link between JA and Fe deficiency responses, we also investigated the overlap of JA-induced genes with those induced by other nutrient deficiencies. Based on a previous microarray analysis by Takehisa et al. (2013), N deficiency-induced genes at 6 h and N, P or K deficiency-induced genes at 24 h were analyzed (Fig. 1b). Among these, K deficiency-induced genes showed notably high overlap with JA-induced genes at both 1–6 h (21 %) and 12 days (17 %), consistent with previous reports that many K deficiency-induced genes are related to JA responses in both rice (Takehisa et al. 2013) and *Arabidopsis* (Armengaud et al. 2004, 2010). N deficiency-induced genes also showed lower but still significant overlap with JA-induced genes at both 1–6 h and 12 days. P deficiency-induced genes showed very small overlap. We further investigated the overlap of JA-induced or -repressed genes with N, P or K-induced or -repressed genes (Online Resource 3). N deficiency-induced genes did not show high overlap with JA-repressed genes, while N deficiency-repressed genes showed significantly overrepresented overlap with JA-induced or -repressed genes. P deficiency-induced or -repressed genes showed significantly overrepresented overlap with JA-induced or -repressed genes only in limited cases. K deficiency-induced genes showed large overlap with JA-repressed genes at 12 days, whereas no JA-induced or -repressed genes were repressed by K deficiency. These results indicate that JA responses are involved in various nutrient deficiencies, and the pattern of the involvement is nutrient-specific, at least for Fe, N, P and K. The high overlap of JA-induced genes at the early stages of deficiency treatment appeared to be characteristic of Fe deficiency. We did not analyze P or K deficiency-induced genes at 6 h, because the numbers of these genes were extremely small (Takehisa et al. 2013).

**Fig. 2:**
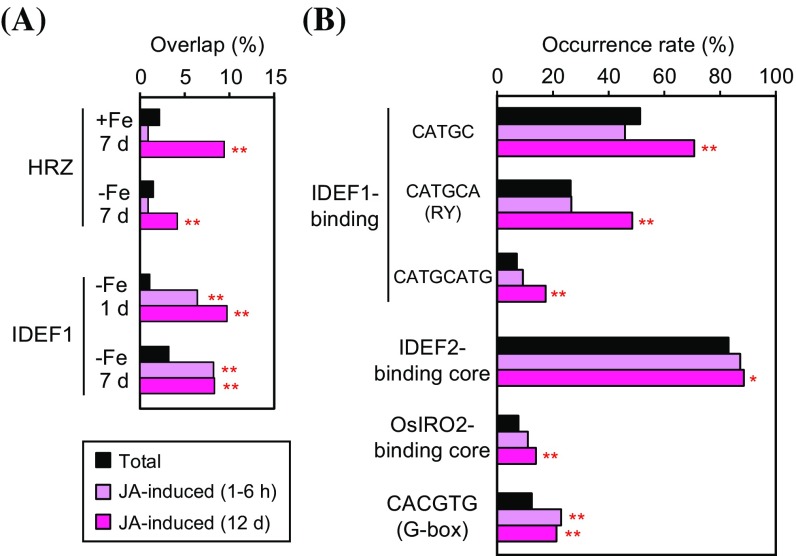
Involvement of *trans*-acting regulators and *cis*-acting elements for Fe deficiency responses in the expression of genes induced by jasmonates (JAs). **a** Overlap of the genes induced by JA treatment after 1–6 h or 12 days (d) with the genes whose expression was induced in *HRZ*-knockdown (HRZ) or *IDEF1*-induced (IDEF1) lines under conditions of Fe sufficiency (+Fe) or deficiency (−Fe) after 1 or 7 days in roots based on previous microarray analyses by Kobayashi et al. (2009, 2013). Overlap was calculated as (the number of the clones showing [induction in *HRZ*-knockdown or *IDEF1*-induced lines] and JA-induction)/(the number of the total clones showing JA-induction). “Total” rate was calculated as (the number of the total clones showing induction in *HRZ*-knockdown or *IDEF1*-induced lines)/(the number of the total clones analyzed). **b** Occurrence rates of *cis*-acting sequences in the genes induced by JA treatment after 1–6 h or 12 days. CATGC, the minimal sequence recognized by IDEF1; CATGCA (RY) and CATGCATG, common binding sequences of IDEF1 and other ABI3/VP1 family transcription factors (Kobayashi et al. 2007); IDEF2-binding core, the minimal sequence recognized by IDEF2 [CA(A/C)G(T/C)(T/C/A)(T/C/A); Ogo et al. 2008]; OsIRO2-binding core, the minimal sequence efficiently recognized by OsIRO2 [CACGTGG; Ogo et al. 2006]; CACGTG (G-box), the binding sequence of many bHLH transcription factors, including MYC2/JIN1, a central regulator of the JA response (Fonseca et al. 2009). The putative promoter regions 500 nucleotides upstream of the 5′ border of the predicted transcription initiation sites were used to search for *cis*-sequences. Occurrence rate was calculated as (the number of the clones possessing the *cis*-sequence and showing JA-induction)/(the number of the total clones showing JA-induction). “Total” rate was calculated as (the number of the total clones possessing the *cis*-sequence)/(the number of the total clones analyzed). *Asterisks* indicate significant overrepresentations compared with the total rates (*P < 0.05; **P < 0.01). No significant underrepresentation was observed

We then investigated the effects of known regulators of Fe deficiency responses on the expression of JA-induced genes. Based on previous microarray analyses (Kobayashi et al. 2009, 2013), the genes induced in the *HRZ*-knockdown or *IDEF1*-induced rice lines, both of which show enhanced Fe deficiency responses (Kobayashi et al. 2007, 2009, 2013), were used for calculating the overlaps with JA-induced genes (Fig. 2a). The genes induced in the *HRZ*-knockdown lines under either Fe-sufficient or -deficient conditions showed significantly higher overlap with JA-induced genes at 12 days, but not at 1–6 h (Fig. 2a). In contrast, significant overrepresentation was observed for the *IDEF1*-induced lines at both 1 and 7 days of Fe deficiency treatments with JA-induced genes at both 1–6 h and 12 days. These results suggest that the late response of the JA pathway is constitutively repressed by OsHRZs, while both the early and late JA responses are activated by IDEF1 under Fe deficiency.

Investigation of *cis*-acting sequences in JA-responsive gene promoters further revealed a relationship between JAs and Fe deficiency responses (Fig. 2b). JA-induced genes at 12 days, but not at 1–6 h, showed significant overrepresentation of *cis*-acting elements mediating Fe deficiency responses, namely, core-binding sequences of IDEF1, IDEF2 and OsIRO2 (Kobayashi et al. 2007; Ogo et al. 2006, 2008; Kakei et al. 2013), suggesting the involvement of these elements and transcription factors during late JA responses. JA-induced gene promoters at both 1–6 h and 12 days also showed overrepresentation of G-box, presumably reflecting a central JA response mediated by MYC2/JIN1 (Fonseca et al. 2009).

Similar analysis of the JA-repressed genes also revealed overrepresentation of the JA-repressed genes at 12 days, but not at 1–6 h, with the *HRZ*-knockdown lines under Fe sufficiency, the *IDEF1*-induced lines at 7 days of Fe deficiency, and IDEF1-binding *cis*-acting sequences (Online Resource 4). These results suggest the involvement of OsHRZs and IDEF1 regulators in both the promotive and repressive pathways of JA responses.

### Several genes involved in JA responses are rapidly induced at the onset of Fe deficiency

**Fig. 3:**
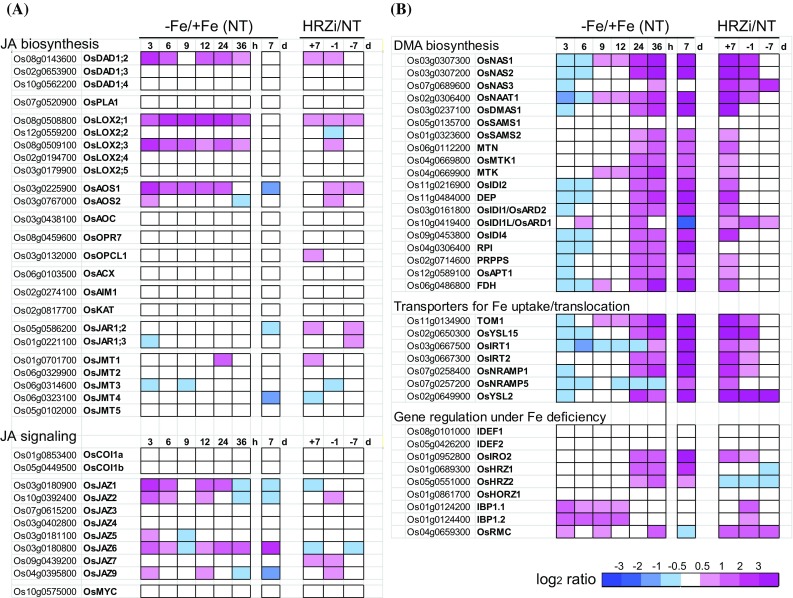
Transcriptional responses of the genes involved in JA and Fe deficiency responses in response to Fe deficiency treatment and *HRZ*-knockdown in roots. **a** Genes involved in JA biosynthesis and signaling. **b** Genes involved in Fe deficiency responses. Expression ratios are shown in the heat maps using a log_2_ scale based on the microarray results as below. Expression ratios at 3–36 h and 7 days (d) of Fe deficiency treatment *versus* control Fe sufficiency treatment in non-transformants [−Fe/+Fe (NT)] are based on previous microarray analyses by Itai et al. (2013) and Ogo et al. (2008), respectively. Expression ratios of *HRZ*-knockdown versus non-transformants (HRZi/NT) at day 7 of Fe sufficiency (+7) or day 1 or 7 of deficiency (−1, −7) are based on the previous microarray analysis by Kobayashi et al. (2013)

To gain further insight into the relationship between JA- and Fe deficiency responses, we investigated the transcriptional responses of the genes involved in JA biosynthesis and signaling in response to early and subsequent Fe deficiency, as well as *HRZ*-knockdown (Fig. 3a). Ten out of those 35 genes were induced very rapidly at 3 h after the onset of Fe deficiency. This induction was sustained for 24 h and then weakened or vanished at 36 h and 7 days (Fig. 3a). Moderately enhanced expression in *HRZ*-knockdown roots compared with non-transformants was also observed for 12 out of those 35 genes under either Fe-sufficient or Fe-deficient conditions (Fig. 3a). These results suggest that JA biosynthesis is enhanced during the very early stages of Fe deficiency in non-transformants and constitutively in *HRZ*-knockdown plants.

Because of the notably rapid induction of some JA response-related genes under early Fe deficiency, we then compared the kinetics of transcriptional expression of these genes with that of genes known to be involved in Fe deficiency responses (Fig. 3a vs b). The majority of genes responsible for Fe uptake and translocation, such as those for DMA biosynthesis and transporters, were strongly induced at 24, 36 h and 7 days of Fe deficiency treatment (Fig. 3b), as reported previously (Itai et al. 2013; Kobayashi et al. 2014). However, these genes began to be induced at no earlier than 9 h (Fig. 3b; Itai et al. 2013), which is in contrast to the markedly earlier induction of JA-related genes at 3 h after the onset of Fe deficiency treatment (Fig. 3a). On the other hand, expression of *IBP1.1*, *IBP1.2* and *OsRMC* began to be induced at 3 h (Fig. 3b). These three genes are putative regulators of rice Fe deficiency responses (Yang et al. 2013; Zhang et al. 2014), and their expression is induced by JA treatment (Jiang et al. 2007; Yoshii et al. 2010).

*HRZ*-knockdown plants hyper-expressed most of the Fe deficiency-inducible genes involved in Fe uptake and translocation mostly under Fe sufficiency (Fig. 3b), as reported previously (Kobayashi et al. 2013). Expression of *IBP1.1*, *IBP1.2* and *OsRMC* genes was also induced in *HRZ*-knockdown plants (Fig. 3b).

### JA concentrations in rice roots tend to be increased at the early stages of Fe deficiency

**Fig. 4:**
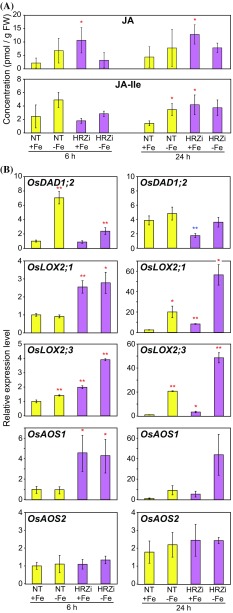
Induction of jasmonates (JAs) during the early stages of Fe deficiency in roots. **a** Endogenous concentrations of jasmonic acid (JA) and jasmonoyl isoleucine (JA-Ile) (means ± SD, n = 3). **b** Transcript levels of *OsDAD1;2*, *OsLOX2;1*, *OsLOX2;3*, *OsAOS1* and *OsAOS2*, genes responsible for JA biosynthesis. Non-transgenic (NT) and *HRZ*-knockdown (HRZi) rice were subjected to Fe sufficiency (+Fe) or deficiency (−Fe) for 6 and 24 h. Roots were harvested and used for quantification of JAs and quantitative RT-PCR. The transcript abundance was normalized against the rice α-2 tubulin transcript level and expressed as a ratio relative to the levels in the NT + Fe at 6 h (means ± SD, n = 3). *Asterisks* indicate significant differences compared with the +Fe NT level at each time point (*P < 0.05; **P < 0.01)

The induction of JA biosynthesis-related genes under early Fe deficiency and in *HRZ*-knockdown plants suggests that the production of JAs is induced in these plants. To prove this hypothesis, we cultured non-transformants and *HRZ*-knockdown plants under Fe deficiency or sufficiency for 6 and 24 h, and quantified endogenous concentrations of JA and JA-Ile, the active form of JA. In non-transgenic roots, concentrations of JA and JA-Ile increased approximately 2–3-fold after Fe deficiency treatment at both 6 and 24 h (Fig. 4a). Particularly, the JA-Ile concentration was 2.4-fold higher in roots subjected to Fe deficiency for 24 h, showing significant increases compared with Fe-sufficiency. In *HRZ*-knockdown roots, concentrations of JAs, especially JA, were much higher than those in non-transformants under Fe sufficiency (Fig. 4a). Concentrations of JAs under Fe deficiency treatment were similar or moderately lower in *HRZ*-knockdown roots than in non-transgenic roots. These results strongly suggested that JA biosynthesis is induced during the early stages of Fe deficiency in non-transgenic roots, while this response is de-repressed in Fe-sufficient *HRZ*-knockdown roots.

We also confirmed the transcriptional responses of JA biosynthetic genes, *OsDAD1;2*, *OsLOX2;1*, *OsLOX2;3*, *OsAOS1* and *OsAOS2*, in these roots using quantitative RT-PCR (Fig. 4b). In non-transgenic roots, *OsDAD1;2* and *OsLOX2;3* showed induction after 6 h of Fe deficiency treatment, while *OsLOX2;1*, *OsLOX2;3*, and *OsAOS1* showed induction after 24 h. *OsAOS2* did not show any induction under Fe deficiency after 6 or 24 h. These transcriptional responses are similar to those observed in previous microarray results, but the induction appears to be slower than the previous results (Fig. 3a). Consistently, expression of *OsNAS2*, *OsIRO2* and *OsYSL2*, representative Fe deficiency-inducible genes involved in Fe utilization, were induced only moderately at 24 h (Online Resource 5), at levels lower than those in the previous microarray results (Fig. 3b; Itai et al. 2013).

The JA biosynthetic genes *OsLOX2;1*, *OsLOX2;3* and *OsAOS1*, but not *OsDAD1;2* and *OsAOS2*, showed much higher expression in *HRZ*-knockdown plants than in non-transformants under both Fe conditions at both 6 and 24 h (Fig. 4b). Strong induction was also observed for *OsNAS2*, *OsIRO2* and *OsYSL2* (Online Resource 5), consistent with previous results (Fig. 3b; Kobayashi et al. 2013). Repressed expression of *OsHRZ2* in *HRZ*-knockdown roots was also confirmed (Online Resource 5). These results confirmed that OsHRZs negatively regulate the expression of some genes involved in the JA signaling pathway, in addition to those involved in Fe utilization.

### JAs act positively and negatively on the expression of typical Fe deficiency-inducible genes depending on Fe nutritional status

**Fig. 5:**
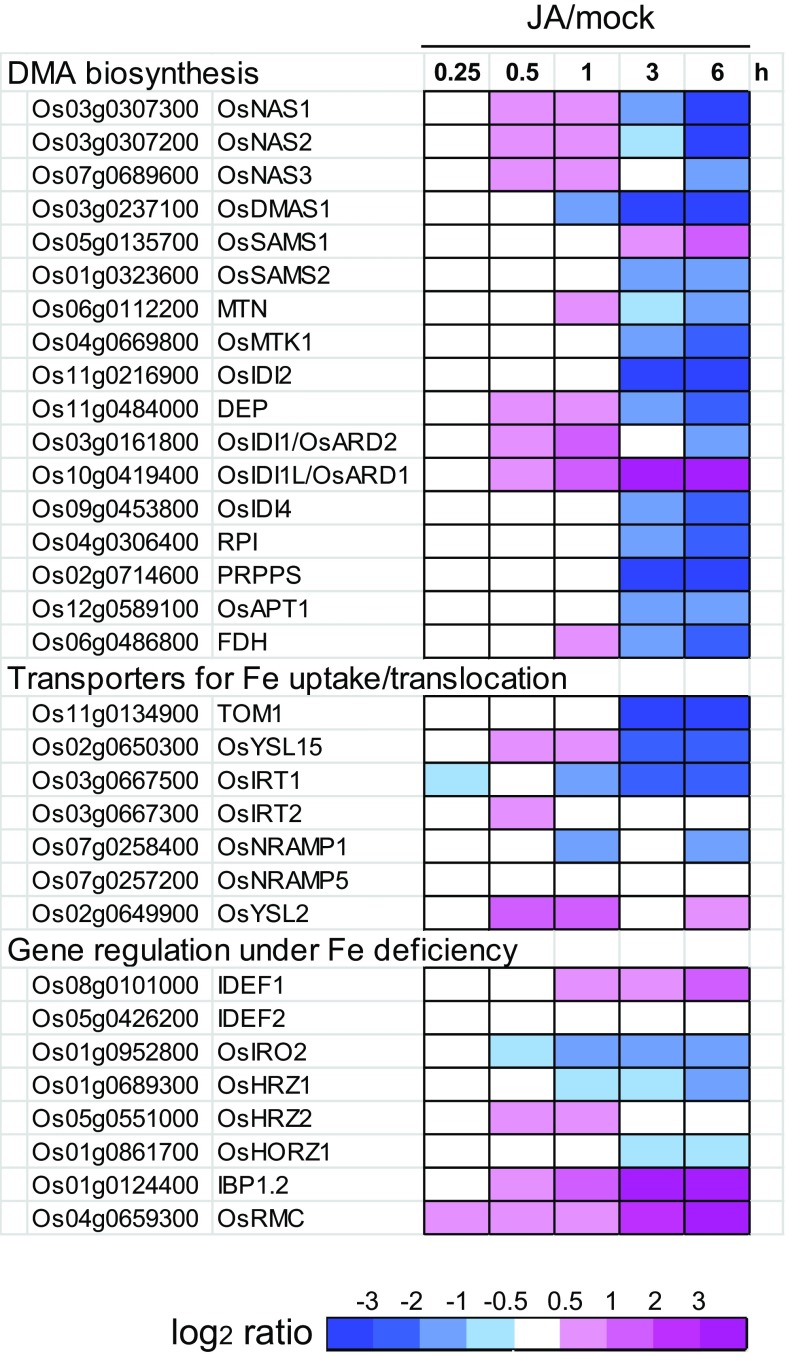
Transcriptional responses of the genes involved in Fe deficiency responses to jasmonic acid (JA) treatment in roots. Expression ratios of 100 μM JA treatment *versus* mock treatment (JA/mock) at 0.25, 0.5, 1, 3, and 6 h are based on the microarray results provided on the RiceXPro website (Sato et al. 2013; http://ricexpro.dna.affrc.go.jp/index.html). The ratios are shown in the heat maps using a log_2_ scale

We then examined whether expression of genes known to be involved in Fe deficiency responses are transcriptionally regulated by JA (Fig. 5). These genes tended to show moderate and transient induction by JA treatment after 0.5–1 h. Subsequently, a majority of these genes showed strong repression at 3–6 h. This repression was particularly dominant among the genes strongly induced under Fe deficiency, such as *OsNAS1*, *OsNAS2*, *OsDMAS1*, and *TOM1*. In contrast, some genes that were not strongly induced under Fe deficiency—including *OsSAMS1*, *OsIDI1L/OsARD1* and *IDEF1*—showed gradual induction by JA treatment after 3–6 h. *IBP1.2* and *OsRMC* showed stronger induction by JA treatment (Fig. 5), consistent with previous reports (Jiang et al. 2007; Yoshii et al. 2010).

**Fig. 6:**
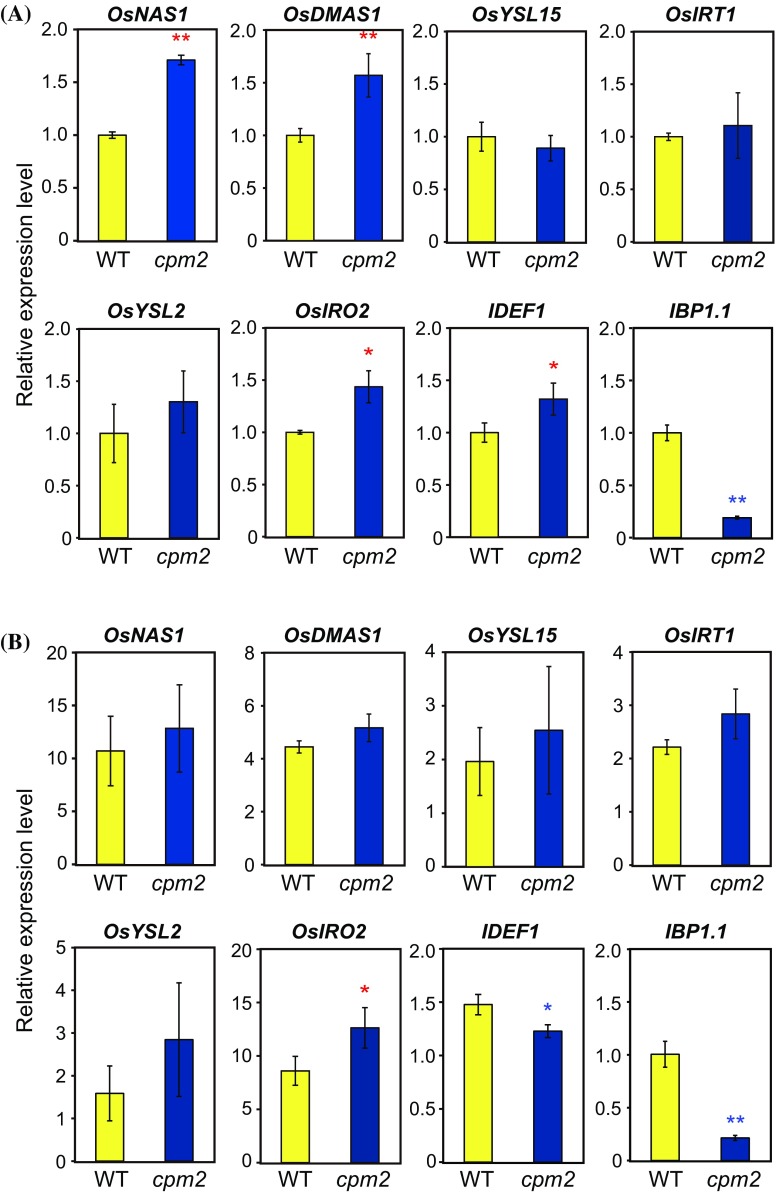
Transcript levels of representative genes involved in Fe deficiency responses in the JA-deficient *cpm2* mutant. Wild-type (WT) and homozygous *cpm2* mutant were subjected to Fe sufficiency (**a**) or deficiency (**b**) for 24 h. Roots were harvested and used for quantitative RT-PCR analysis. The transcript abundance was normalized against the rice α-2 tubulin transcript level and expressed as a ratio relative to the levels in the Fe-sufficient WT plants (means ± SD, n = 3). *Asterisks* indicate significant differences compared with the WT level at each condition (*P < 0.05; **P < 0.01)

We further investigated the effect of endogenous JAs on Fe deficiency responses. For this purpose, we utilized the homozygous *cpm2* mutant, in which concentration of endogenous JAs is extremely low because of a mutation in the *OsAOC* gene, encoding a key enzyme in JAs biosynthesis (Riemann et al. 2013). We measured the transcript levels of representative genes involved in Fe deficiency responses in wild-type and the *cpm2* mutants cultured under Fe deficiency or sufficiency for 24 h (Fig. 6). Under Fe sufficiency, three Fe deficiency-inducible genes, *OsNAS1*, *OsDMAS1* and *OsIRO2,* showed higher expression in the *cpm2* mutants than in wild-type (Fig. 6a), indicative of negative regulation of these genes by the JA signaling. However, this regulation was diminished after 24 h of Fe deficiency, except for *OsIRO2* (Fig. 6b). Three other Fe deficiency-inducible genes, *OsYSL15*, *OsIRT1* and *OsYSL2,* showed similar level of expression in wild-type and the *cpm2* mutants under both Fe conditions (Fig. 6a, b). Expression level of *IDEF1* was higher in the *cpm2* mutants under Fe sufficiency, but was lower under Fe deficiency, compared to wild-type (Fig. 6a, b). Expression of *IBP1.1* was strongly repressed in the *cpm2* mutants in both Fe conditions (Fig. 6a, b), consistent with the JA-inducible nature of this gene (Yoshii et al. 2010). We also confirmed similar repression in expression of *OsLOX2;1* and *OsAOS2*, JA biosynthetic genes induced by JA treatment, in the *cpm2* mutants (Online Resource 6). These results indicate that JAs repress a subset of Fe deficiency-inducible genes under Fe-sufficient conditions, but this repression is partly canceled at an early stage of Fe deficiency.

We also investigated tolerance of the *cpm2* mutant to Fe deficiency. During hydroponic culture under Fe-deficient condition, the *cpm2* mutant showed similar but slightly lower chlorophyll level of the newest leaves, an indicator of Fe deficiency symptoms, compared to wild-type counterpart (Online Resource 7). This result confirmed that enhanced expression of some genes involved in Fe uptake and translocation in Fe-sufficient *cpm2* mutant does not result in enhanced tolerance to Fe deficiency.

## Discussion

Involvement of JAs in Fe deficiency responses has been reported only in *Arabidopsis*, in which administration of methyl-JA exerts negative effects on the expression of the typical Fe deficiency-induced genes *IRT1*, *FRO2* and *FIT* (Maurer et al. 2011). This report suggested a generally repressive role for JAs in Fe deficiency responses (Hindt and Guerinot 2012). However, we have noted the possibility of positive effects of JAs on Fe deficiency responses, based on the putatively positive regulation of rice Fe deficiency responses by the JA-induced genes *IBP1.1*, *IBP1.2* and *OsRMC* (Yang et al. 2013; Zhang et al. 2014). A positive effect of JAs on Fe deficiency responses has also been suggested in maize intercropped with peanut, which induces both Fe deficiency responses and protein-level expression of key enzymes involved in JA biosynthesis (Xiong et al. 2013a, b). In the present report, we identified the activation of JA signaling during the early stages of Fe deficiency responses in rice roots. We also found that JAs repress the expression of some genes involved in Fe deficiency responses under Fe sufficiency, but this repression is partly canceled at early stages of Fe deficiency.

When Fe availability decreases, root cells perceive unidentified Fe deficiency signals and start inducing genes responsible for Fe uptake and translocation. This induction was not obvious before 9 h from the onset of Fe deficiency (Fig. 3b; Itai et al. 2013). We identified that the same plants induce many genes involved in JA biosynthesis and responses during the earlier stages, no later than 3 h (Figs. 1, 3a). Precedence of the JA responses and increase in the concentration of JAs in Fe deficiency-treated rice roots were also suggested in another culture (Fig. 4, Online Resource 5), although induction of the JA-related and Fe acquisition-related genes was slower in this culture compared to a previous microarray analysis (Fig. 3; Itai et al. 2013). Presumably, the plants in the present experiment experienced less-severe Fe deficiency due to differences in growth conditions, such as plant size. Nevertheless, our results suggested that rapid production of JAs and activation of the JA signaling pathway precede the induction of typical Fe deficiency-inducible genes at the onset of Fe deficiency. Thus, the transient production of JAs and activation of the JA pathway might play important roles during the very early stages of Fe deficiency responses.

This hypothesis is supported by the expressional response of Fe deficiency-inducible genes to JA treatment and endogenous JA deficiency. Many of these genes were transiently induced but subsequently repressed by JA administration (Fig. 5). The JA-deficient *cpm2* mutant showed higher expression of Fe deficiency-inducible genes, *OsNAS1*, *OsDMAS1* and *OsIRO2,* under Fe sufficiency (Fig. 6a). Thus, these genes are thought to be repressed by JAs, similarly to *Arabidopsis* Fe deficiency-inducible genes (Maurer et al. 2011), at normal Fe condition. However, this repression was canceled under early Fe deficiency, except for *OsIRO2* (Fig. 6b). These results indicate that the repressive effect of JAs in rice roots is dependent on Fe nutritional status, and is mitigated at early stages of Fe deficiency. This might be due to promotive effect of transiently synthesized JAs at very early stages of Fe deficiency, which might counteract the constitutive and repressive effect of JAs. The JA pathway might fine-tune the expression kinetics of Fe deficiency-induced genes. In *Arabidopsis*, repression of typical Fe deficiency-induced genes was observed after methyl-JA treatment for 6 h or 3 days (Maurer et al. 2011). Analysis of earlier responses to JA administration is necessary to confirm whether JA exhibits promotive effects in *Arabidopsis* and other plant species similar to those in rice.

**Fig. 7:**
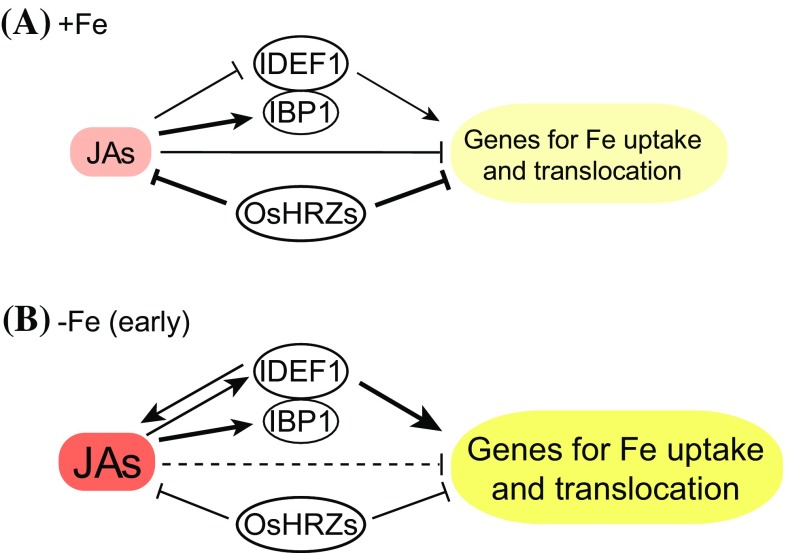
Summary of the regulation of the genes involved in Fe uptake and translocation by JAs, IDEF1 and OsHRZs in rice roots. **a** Fe-sufficient conditions. **b** Early stages of Fe-deficient conditions. Expressional regulation identified previously (Kobayashi et al. 2009, 2013, 2014) and in the present report is depicted. The thickness of the lines indicates the relative strength of the regulation. The majority of genes responsible for Fe uptake and translocation are induced in response to Fe deficiency (Itai et al. 2013; Kobayashi et al. 2014). Prior to this induction, concentrations of JA and JA-Ile tend to be increased, which might be responsible for mitigation of constitutive repression of the genes involved in Fe uptake and translocation by JAs (*dashed line* in (**b**)). The transcription factor IDEF1 positively regulates major genes involved in Fe uptake and translocation, and this regulation is intensified during early stages of Fe-deficient conditions (Kobayashi et al. 2009). Conversely, the ubiquitin ligase OsHRZs negatively regulate major genes involved in Fe uptake and translocation, and this regulation become less obvious with the progression of Fe deficiency (Kobayashi et al. 2013). IBP1 binds to IDEF1 and is supposed to enhance the stability and function of IDEF1 (Zhang et al. 2014)

The involvement of JAs in rice Fe deficiency responses is summarized in Fig. 7. Our results indicate that the effects of JAs are dependent on Fe status, and also on IDEF1 and OsHRZs, which are positive and negative regulators of Fe deficiency responses, respectively (Kobayashi et al. 2007, 2009, 2013, 2014). Our correlation analysis suggested that JA-responsive genes are positively regulated by IDEF1 at both 1 and 7 days of Fe deficiency (Fig. 2a), which correspond to early and subsequent stages of IDEF1-mediated Fe deficiency responses, respectively (Kobayashi et al. 2009). Because IDEF1 is especially important for early Fe deficiency responses (Kobayashi et al. 2009), the JA pathway might be involved in triggering these responses in roots. In addition, *IDEF1* and its possible activator *IBP1.2* showed gradual induction by JA treatment over 1–6 h, in contrast to repression of typical Fe deficiency-induced genes by JA treatment after 3–6 h (Fig. 5). Although expression of *IBP1.1*, a close homolog of *IBP1.2*, is not available in this database, a previous report indicated that expression of *IBP1.1* (Os01g0124200) and *IBP1.2* (Os01g0124400) is similarly induced by JA treatment at 12 days (Yoshii et al. 2010). IBP1.1 and IBP1.2 interact with IDEF1, putatively supporting the function of IDEF1 by preventing its proteasomal degradation (Zhang et al. 2014). Expressional analysis in the *cpm2* mutant also indicated that expression of *IDEF1* and *IBP1.1* is induced by JAs under early Fe deficiency (Fig. 6b). Under Fe sufficiency, expression of *IBP1.1* was similar, but *IDEF1* expression showed the opposite tendency (Fig. 6a). Taken together, positive interaction between the IDEF1 pathway and JA signaling occurs during the early stages of Fe deficiency (Fig. 7b).

Our results also revealed that OsHRZ ubiquitin ligases suppress the JA pathway, especially under Fe sufficiency (Fig. 7). Expression analysis of *HRZ*-knockdown lines indicated that OsHRZs negatively regulate the expression of late JA-responsive genes and several genes involved in JA biosynthesis and signaling (Figs. 2a, 3a). In addition, *HRZ*-knockdown roots accumulated more JAs than did non-transformants under Fe sufficiency (Fig. 4a). OsHRZs and their *Arabidopsis* homolog BTS are Fe- and Zn-binding ubiquitin ligases that negatively regulate Fe deficiency responses (Long et al. 2010; Kobayashi et al. 2013; Selote et al. 2015), possibly preventing excessive Fe uptake and translocation. Repression of the JA pathway by OsHRZs revealed in the present study (Figs. 2a, 3a, 4) suggests mitigation of this Fe-limiting function of HRZs/BTS by preventing JA-mediated repression of typical genes.

Although OsHRZs constitutively repress Fe uptake and translocation, as evidenced by the Fe-accumulating phenotype of *HRZ*-knockdown lines irrespective of Fe nutritional status (Kobayashi et al. 2013), several lines of evidence suggest that HRZs/BTS alter or modify their functions in response to Fe availability. Expression of HRZs/BTS is transcriptionally induced under Fe deficiency, although their induction levels are somewhat lower than those of typical Fe deficiency-inducible genes involved in Fe homeostasis (Fig. 3b; Long et al. 2010; Kobayashi et al. 2013). In addition, BTS protein is destabilized under Fe-rich conditions in vitro (Selote et al. 2015), suggesting preferential roles for HRZs/BTS under Fe-deficient conditions. In contrast to these observations, the transcriptional profiles of *HRZ*-knockdown rice lines indicated that OsHRZs repress the expression of major genes involved in Fe uptake and translocation preferentially under Fe-sufficient conditions (Fig. 3b; Kobayashi et al. 2013). In the present study, we found higher concentrations of JAs in *HRZ*-knockdown lines compared with non-transformants only under Fe sufficiency (Fig. 4a), although the transcript levels of *OsLOX2;1*, *OsLOX2;3* and *OsAOS1*, genes responsible for JA biosynthesis, were markedly higher in *HRZ*-knockdown lines under both Fe-sufficient and -deficient conditions (Fig. 4b). This phenomenon might be due to failure of further induction of rate-limiting enzyme(s) for JA biosynthesis in *HRZ*-knockdown lines, or a feedback mechanism for limiting JA concentrations under Fe deficiency. The preferential effect of OsHRZs on JA concentrations under Fe-sufficient conditions coincides with their predominant regulation of Fe acquisition-related genes under Fe sufficiency (Fig. 3b; Kobayashi et al. 2013). Interestingly, accumulation of endogenous JA-Ile was less dominant than that of JA in Fe-sufficient *HRZ*-knockdown roots (Fig. 4a), although the expression of *OsJAR1;2*, encoding a putative enzyme for JA-Ile biosynthesis from JA (Hirano et al. 2008), was enhanced in Fe-sufficient *HRZ*-knockdown roots (Fig. 3a). Possibly, protein degradation or inactivation of OsJAR1;2 or other biosynthetic genes for JA-Ile might occur in Fe-sufficient *HRZ*-knockdown roots to prevent excessive JA signaling.

The increased concentrations of JAs in *HRZ*-knockdown plants under normal Fe supply also suggest altered responses of these plants to various biotic and abiotic stresses regulated by the JA pathways (Fonseca et al. 2009; Vleesschauwer et al. 2013). Indeed, close homologs of HRZs/BTS in *Lotus japonicus* and tobacco regulate the responses to bacterial and viral infections, respectively (Shimomura et al. 2006; Yamaji et al. 2010). HRZs/BTS and their homologs are widely distributed in the plant kingdom (Urzica et al. 2012; Kobayashi et al. 2013), and might play significant roles linking Fe deficiency responses, JA responses and other stress responses in plants.

In conclusion, we clarified that JA biosynthesis and signaling pathways are rapidly induced in rice roots in response to very early Fe deficiency, and are constitutively activated by *HRZ*-knockdown. JAs negatively regulate some Fe deficiency-inducible genes under Fe-sufficient conditions, but this regulation is partly canceled under early Fe deficiency. The JA signaling in early Fe deficiency responses is partly regulated by IDEF1 and OsHRZs. Identification of the molecular mechanisms by which IDEF1 and OsHRZs sense and transmit Fe deficiency signaling will be key to clarifying the corresponding signaling pathways.

## Electronic supplementary material

Below is the link to the electronic supplementary material.

Supplementary material 1 (DOCX 220 kb)
