# Supplementary material for: Jasmonate signaling is activated in the very early stages of iron deficiency responses in rice roots
Source: Plant Mol Biol. 2016 May 3;91:533–47. doi: 10.1007/s11103-016-0486-3 (PMC4914535; doi:10.1007/s11103-016-0486-3)
Supplement: Supplementary file 1 — Supplementary material 1 (DOCX 220 kb) [file 11103_2016_486_MOESM1_ESM.docx]

**Online Resource for:**

**Jasmonate signaling is activated in the very early stages of iron deficiency responses in rice roots**

**Journal:** Plant Molecular Biology

**Authors:** Takanori Kobayashi^1,2,*^, Reiko Nakanishi Itai^3^, Takeshi Senoura^2^, Takaya Oikawa^4^, Yasuhiro Ishimaru^4^, Minoru Ueda^4^, Hiromi Nakanishi^3^, and Naoko K. Nishizawa^2^

**Affiliations:**

^1^Japan Science and Technology Agency, PRESTO, 4-1-8 Honcho, Kawaguchi, Saitama 332-0012, Japan.

^2^Research Institute for Bioresources and Biotechnology, Ishikawa Prefectural University, 1-308 Suematsu, Nonoichi, Ishikawa 921-8836, Japan.

^3^Graduate School of Agricultural and Life Sciences, The University of Tokyo, 1-1-1 Yayoi, Bunkyo-ku, Tokyo 113-8657, Japan.

^4^Graduate School of Science, Tohoku University, 6-3 Aramaki-aza Aoba, Aoba-ku, Sendai 980-8578, Japan.

**^*^Correspondence to:** Takanori Kobayashi

Tel: +81 76 259 0579; Fax: +81 76 227 7557; E-mail: [abkoba@ishikawa-pu.ac.jp](mailto:abkoba@ishikawa-pu.ac.jp)

**Online Resource 1.** Primers used for quantitative RT-PCR.

| **Primer name** | **Sequence** |
| --- | --- |
| *OsDAD1;2* forward | 5’-ACGTCAAGCAGCTCTACATCAG-3’ |
| *OsDAD1;2* reverse | 5’-AAATATTTCTTGACCATCAGGAGG-3’ |
| *OsLOX2;1* forward | 5’-AACGCTCCAAAACTACTTGC-3’ |
| *OsLOX2;1* reverse | 5’-ACATTAAACATTGTGATACCTTGAG-3’ |
| *OsLOX2;3* forward | 5’-TGGGAGGACATCTACTTGC-3’ |
| *OsLOX2;3* reverse | 5’-AACATCAACAACAACCACTTC-3’ |
| *OsAOS1* forward | 5’-GCCGGCCATCACTTCAG-3’ |
| *OsAOS1* reverse | 5’-GTTTAAAGACACTGTCAGCCCTG-3’ |
| *OsAOS2* forward | 5’-CCACCGCCGGTCAAAG-3’ |
| *OsAOS2* reverse | 5’-AACTCCGTATCCGTACAAGCTG-3’ |
| *OsNAS1* forward | 5’-GTCTAACAGCCGGACGATCGAAAGG-3’ |
| *OsNAS1* reverse | 5’-TTTCTCACTGTCATACACAGATGGC-3’ |
| *OsDMAS1* forward | 5’-GCCGGCATCCCGCAGCGGAAGATCA-3’ |
| *OsDMAS1* reverse | 5’-CTCTCTCTCTCGCACGTGCTAGCGT-3' |
| *OsYSL15* forward | 5’-CACCCTGGTGAAGCAGCTGGTGCTC-3’ |
| *OsYSL15* reverse | 5’-CGGCCATCGCCGTCGGCAGCGGCAC-3’ |
| *OsIRT1* forward | 5’-CGTCTTCTTCTTCTCCACCACGAC-3’ |
| *OsIRT1* reverse | 5’-GCAGCTGATGATCGAGTCTGACC-3’ |
| *OsYSL2* forward | 5’-TCTGCTGGCTTCTTTGCATTTTCTG-3’ |
| *OsYSL2* reverse | 5’-ACCATGTCGAACTCAGCATCCAGGA-3' |
| *OsIRO2* forward | 5’-CCGGCGGATCCCGCTCCCAC-3' |
| *OsIRO2* reverse | 5’-CGTCGTCGTCAGCTCCTTCT-3’ |
| *IDEF1* | TaqMan Gene Expression Assays Os03570590_m1 |
| *IBP1.1* | TaqMan Gene Expression Assays Os03470892_g1 |
| *a-2 tubulin* | TaqMan Gene Expression Assays Os03562997_mH |
| *OsNAS2* forward | 5’-TGAGTGCGTGCATAGTAATCCTGGC-3’ |
| *OsNAS2* reverse | 5’-CAGACGGTCACAAACACCTCTTGC-3’ |
| *OsHRZ2* | TaqMan Gene Expression Assays Os03496742_g1 |


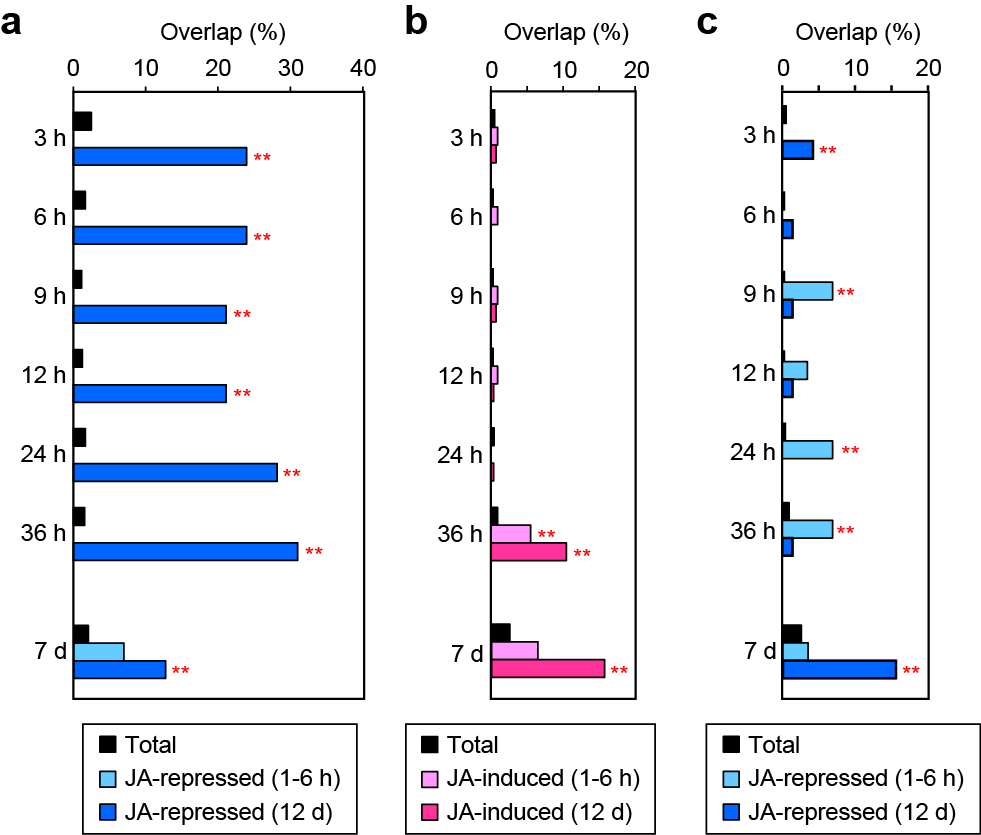


**Online Resource 2.** Overlap of the genes induced or repressed by jasmonates (JAs) with Fe deficiency-induced or -repressed genes. (a) JA-repressed genes after 1–6 h or 12 d with the Fe deficiency-induced genes. (b) JA-induced genes after 1–6 h or 12 d with the Fe deficiency-repressed genes. (c) JA-repressed genes after 1–6 h or 12 d with the Fe deficiency-repressed genes. Genes induced or repressed by JA treatment after 1–6 h and 12 d were analyzed based on previous reports by Yoshii et al. (2010) and Seo et al. (2011). Fe deficiency-induced or -repressed genes at 3–36 h and 7 d are based on previous microarray analyses by Itai et al. (2013) and Ogo et al. (2008), respectively. Overlap was calculated as (the number of the clones showing Fe deficiency-induction or -repression and JA-induction or -repression)/(the number of the total clones showing JA-induction or -repression). “Total” rate was calculated as (the number of the total clones showing Fe deficiency-induction or -repression)/(the number of the total clones analyzed). Asterisks indicate significant overrepresentations compared with the Total rates (**, *P* < 0.01). No significant underrepresentation was observed.

**
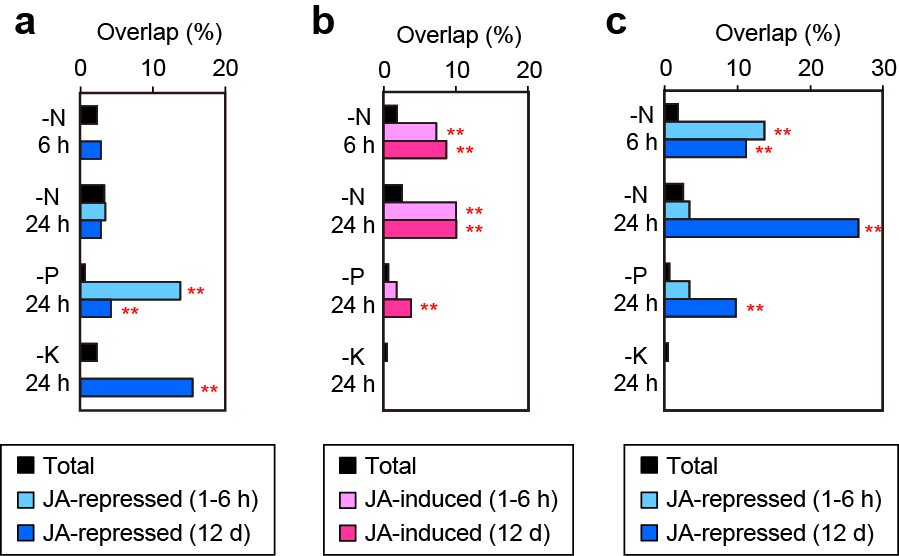
**

**Online Resource 3.** Overlap of the genes induced or repressed by jasmonates (JAs) with nutrient deficiency-induced or -repressed genes. (a) JA-repressed genes after 1–6 h or 12 d with the nutrient deficiency-induced genes. (b) JA-induced genes after 1–6 h or 12 d with the nutrient deficiency-repressed genes. (c) JA-repressed genes after 1–6 h or 12 d with the nutrient deficiency-repressed genes. Genes induced or repressed by JA treatment after 1–6 h and 12 d were analyzed based on previous reports by Yoshii et al. (2010) and Seo et al. (2011). N, P or K deficiency-induced or -repressed genes at 6 or 24 h are based on previous microarray analysis by Takehisa et al. (2013). Overlap was calculated as (the number of the clones showing nutrient deficiency-induction or -repression and JA-induction or -repression)/(the number of the total clones showing JA-induction or -repression). “Total” rate was calculated as (the number of the total clones showing nutrient deficiency-induction or -repression)/(the number of the total clones analyzed). Asterisks indicate significant overrepresentations compared with the Total rates (**, *P* < 0.01). No significant underrepresentation was observed.

**
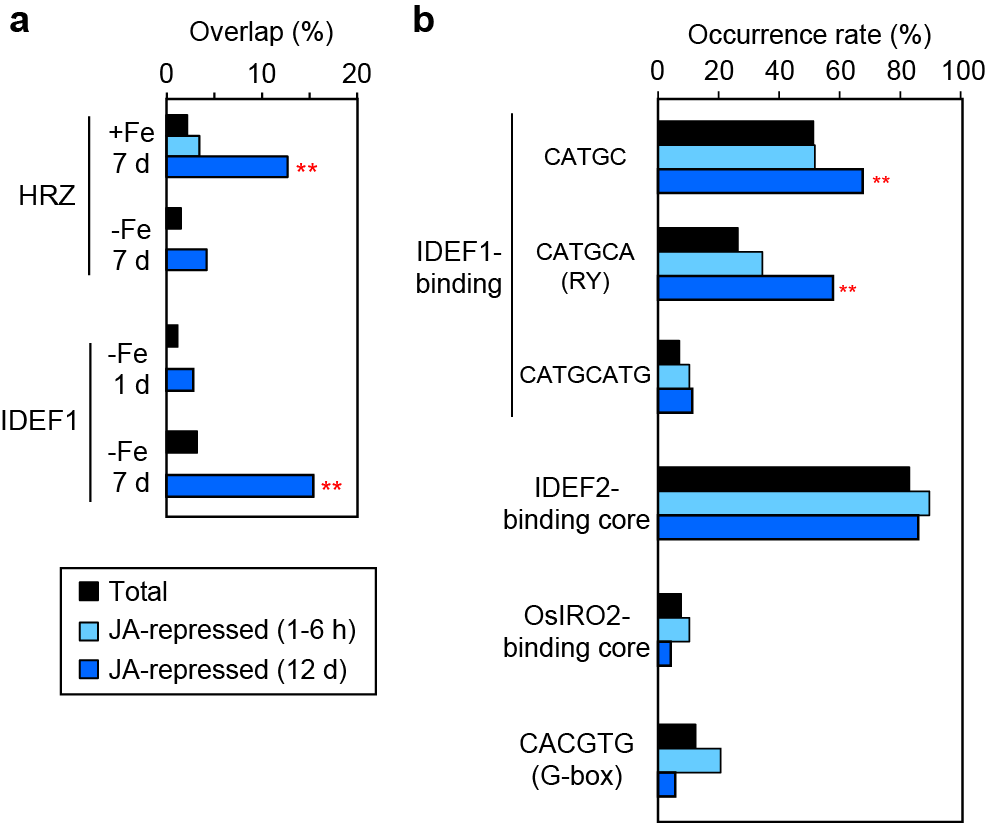
**

**Online Resource 4.** Involvement of *trans*-acting regulators and *cis*-acting elements for Fe deficiency responses in the expression of genes repressed by jasmonates (JAs). (a) Overlap of the genes repressed by JA treatment after 1–6 h or 12 d with those whose expression was induced in *HRZ*-knockdown (HRZ) or *IDEF1*-induced (IDEF1) lines under Fe sufficiency (+Fe) or deficiency (-Fe) after 1 or 7 d based on previous microarray analyses by Kobayashi et al. (2009, 2013). Overlap was calculated as (the number of the clones showing [induction in *HRZ*-knockdown or *IDEF1*-induced lines] and JA-repression)/(the number of the total clones showing JA-repression). “Total” rate was calculated as (the number of the total clones showing induction in *HRZ*-knockdown or *IDEF1*-induced lines)/(the number of the total clones analyzed). (b) Occurrence rates of *cis*-acting sequences in the genes repressed by JA treatment after 1–6 h or 12 d. CATGC, the minimal sequence recognized by IDEF1; CATGCA (RY) and CATGCATG, common binding sequences of IDEF1 and other ABI3/VP1 family transcription factors (Kobayashi et al. 2007); IDEF2-binding core, the minimal sequence recognized by IDEF2 [CA(A/C)G(T/C)(T/C/A)(T/C/A); Ogo et al. 2008]; OsIRO2-binding core, the minimal sequence efficiently recognized by OsIRO2 [CACGTGG; Ogo et al. 2006]; CACGTG (G-box), the binding sequence of many bHLH transcription factors, including MYC2/JIN1, a central regulator of the JA response (Fonseca et al. 2009). The putative promoter regions 500 nucleotides upstream of the 5' border of the predicted transcription initiation sites were used to search for *cis*-sequences. Occurrence rate was calculated as (the number of the clones possessing the *cis*-sequence and showing JA-repression)/(the number of the total clones showing JA-repression). “Total” rate was calculated as (the number of the total clones possessing the *cis*-sequence)/(the number of the total clones analyzed). Asterisks indicate significant overrepresentations compared with the Total rates (*, *P* < 0.05; **, *P* < 0.01). No significant underrepresentation was observed.


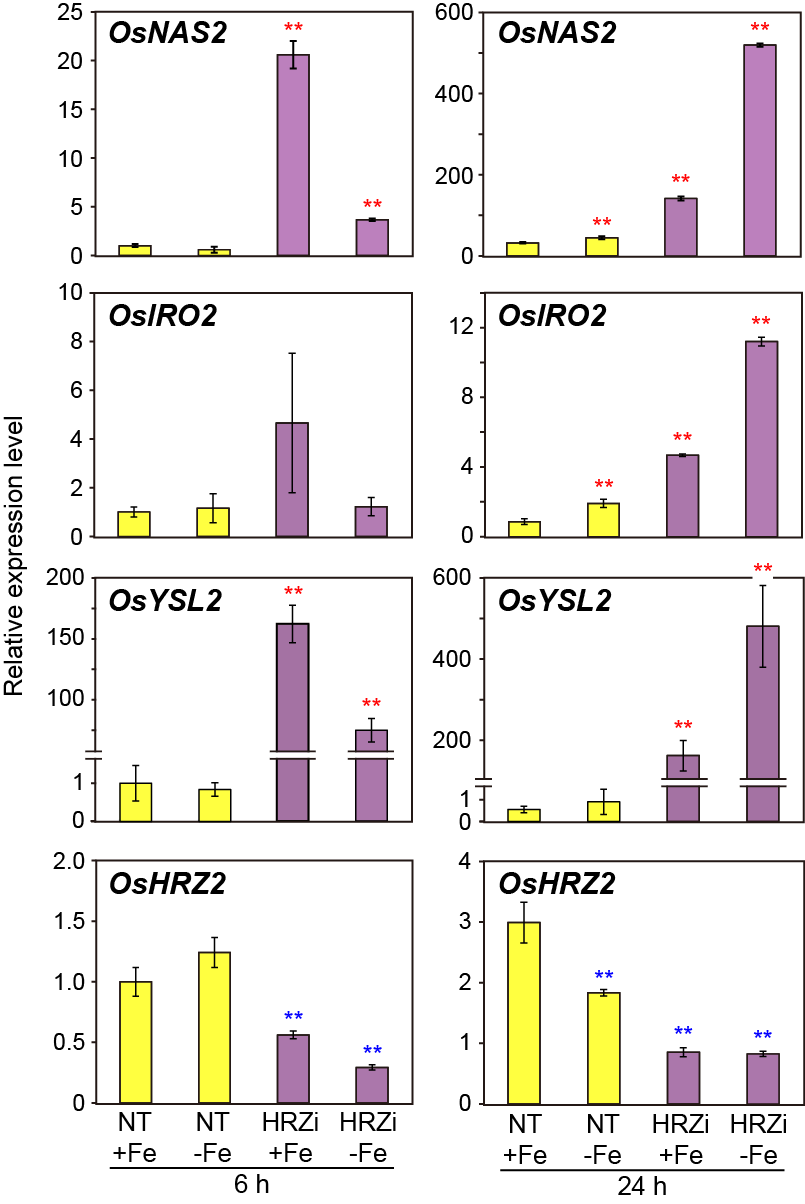


**Online Resource 5.** Transcript levels of the Fe deficiency-inducible genes *OsNAS2*, *OsIRO2*, *OsYSL2*, and *OsHRZ2* during the early stages of Fe deficiency. The plants used for the analysis were identical to those in **Fig. 4**. Non-transgenic (NT) and *HRZ-*knockdown (HRZi) rice were subjected to Fe sufficiency (+Fe) or deficiency (-Fe) for 6 and 24 h. Roots were harvested and used for quantitative RT-PCR analysis. The transcript abundance was normalized against the rice α-2 tubulin transcript level and expressed as a ratio relative to the levels in the NT +Fe after 6 h (means ± SD, n = 3). Asterisks indicate significant differences compared with the +Fe NT level at each time point (*, *P* < 0.05; **, *P* < 0.01).


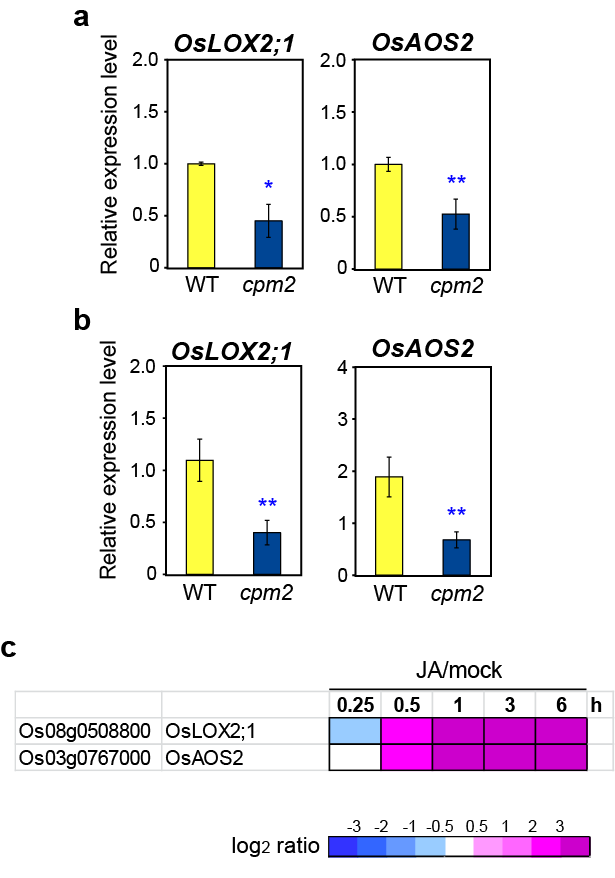


**Online Resource 6.** Transcript levels of *OsLOX2;1* and *OsAOS2* in the JA-deficient *cpm2* mutant. (a, b) Wild-type (WT) and homozygous *cpm2* mutant were subjected to Fe sufficiency (a) or deficiency (b) for 24 h. Roots were harvested and used for quantitative RT-PCR analysis. The transcript abundance was normalized against the rice α-2 tubulin transcript level and expressed as a ratio relative to the levels in the Fe-sufficient WT plants (means ± SD, n = 3). Asterisks indicate significant differences compared with the WT level at each condition (*, *P* < 0.05; **, *P* < 0.01). (c) Transcriptional responses of *OsLOX2;1* and *OsAOS2* to jasmonic acid (JA) treatment in roots. Expression ratios of 100 μM JA treatment *versus* mock treatment (JA/mock) at 0.25, 0.5, 1, 3, and 6 h are based on the microarray results provided on the RiceXPro website (Sato et al. 2013; <http://ricexpro.dna.affrc.go.jp/index.html>). The ratios are shown in the heat maps using a log_2_ scale.


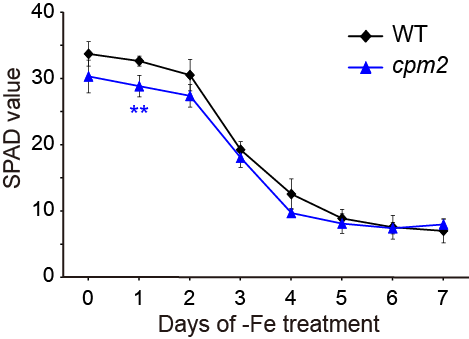


**Online Resource 7.** Tolerance of the JA-deficient *cpm2* mutant to Fe deficiency. SPAD values (indicative of relative chlorophyll contents) of the newest leaves of the wild-type (WT) and homozygous *cpm2* mutant were measured during the Fe-deficiency treatment in hydroponics (means ± SD, n = 4). Asterisks indicate significant differences compared with the WT level at each time point (**, *P* < 0.01).
